# Iron and Sphingolipids as Common Players of (Mal)Adaptation to Hypoxia in Pulmonary Diseases

**DOI:** 10.3390/ijms21010307

**Published:** 2020-01-02

**Authors:** Sara Ottolenghi, Aida Zulueta, Anna Caretti

**Affiliations:** Biochemistry and Molecular Biology Lab., Health Sciences Department, University of Milan, Via A. di Rudinì, 8, 20142 Milan, Italy; aida.zulueta@unimi.it (A.Z.); anna.caretti@unimi.it (A.C.)

**Keywords:** hypoxia, iron, sphingolipids, hepcidin, ceramide, adaptation, COPD, Cystic Fibrosis, ARDS

## Abstract

Hypoxia, or lack of oxygen, can occur in both physiological (high altitude) and pathological conditions (respiratory diseases). In this narrative review, we introduce high altitude pulmonary edema (HAPE), acute respiratory distress syndrome (ARDS), Chronic Obstructive Pulmonary Disease (COPD), and Cystic Fibrosis (CF) as examples of maladaptation to hypoxia, and highlight some of the potential mechanisms influencing the prognosis of the affected patients. Among the specific pathways modulated in response to hypoxia, iron metabolism has been widely explored in recent years. Recent evidence emphasizes hepcidin as highly involved in the compensatory response to hypoxia in healthy subjects. A less investigated field in the adaptation to hypoxia is the sphingolipid (SPL) metabolism, especially through Ceramide and sphingosine 1 phosphate. Both individually and in concert, iron and SPL are active players of the (mal)adaptation to physiological hypoxia, which can result in the pathological HAPE. Our aim is to identify some pathways and/or markers involved in the physiological adaptation to low atmospheric pressures (high altitudes) that could be involved in pathological adaptation to hypoxia as it occurs in pulmonary inflammatory diseases. Hepcidin, Cer, S1P, and their interplay in hypoxia are raising growing interest both as prognostic factors and therapeutical targets.

## 1. Introduction

Hypoxia is characterized by a decrease in the partial pressure of oxygen in the blood and a consequent decreased supply of oxygen to the tissues. This condition can occur both in para-physiological conditions (subjects exposed to low atmospheric pressures, at high altitudes) and as a consequence of respiratory pathologies such as Acute Respiratory Distress Syndrome (ARDS), Chronic Obstructive Pulmonary Disease (COPD), and Cystic Fibrosis (CF). In such pathologies, as long as in the maladaptation to high altitude which results in High Altitude Pulmonary Edema (HAPE), both the hypoxia-induced and inflammatory pathways play a key role in determining the disease progression. Inflammation is one of the main mechanisms influencing the maladaptation to hypoxia, especially when causing a chronic decrease in hemoglobin (Hb) production, a phenomenon known as anemia of inflammation.

Alveolar hypoxia itself is a strong inducer of pulmonary inflammation. It is well recognized that it generates reactive oxygen species (ROS) and that it promotes nuclear factor kappa B (NFĸB) activation, which in turn upregulates numerous pro-inflammatory cytokines such as IL-1, IL-6, and TNFα [1]. Excess ROS production, enhanced by the presence of unbound iron through the Fenton reaction [2], results in lipid peroxidation [3] and in the decrease in vasodilator NO pool [2]. Chen and colleagues observed that alveolar hypoxia triggers pulmonary vasoconstriction and systemic inflammation by activating alveolar macrophages that in turn promote the ET-1, iNOS, and NO/cGMP pathways [4]. In a model of inflammation following aspiration-induced lung injury, HIF-1α robust increase upregulates the release of pro-inflammatory cytokines depending on NF-κB signaling in type 2 alveolar epithelial cells [5].

In this narrative review, we mention respiratory pathologies as examples of either acute (HAPE and ARDS) or chronic (CF and COPD) hypoxia, and highlight some of the potential mechanisms influencing the prognosis of patients. We will focus on the roles of iron and sphingolipid (SPL) metabolism in determining the adaptation or maladaptation to hypoxia, either physiological or pathological. Upon an overview of the role of iron and SPL in hypoxia, we started by reviewing studies investigating the effects of hypoxia on healthy subjects at high altitude. Then, we looked for studies assessing the most relevant markers that could correlate with prognosis and therapy in hypoxic patients. Our aim is to identify pathways and/or markers involved in the para-physiological adaptation to low atmospheric pressures (high altitudes) that could play a key role in pathological adaptation to hypoxia conditions such as HAPE, or respiratory pathologies such as ARDS, COPD, and CF.

## 2. Maladaptation to Hypoxia: The Case of HAPE and Respiratory Pathologies

### 2.1. High-Altitude Pulmonary Edema 

HAPE is a potentially fatal condition, occurring at altitudes greater than 3,000 m. It affects rapidly ascending, non-acclimatized healthy individuals and it is the leading cause of death due to high-altitude illnesses [6]. At 3000 m, the reduction in mitochondrial PO2 due to the hypobaric hypoxia induces dysfunctions into the electron transport chain that fails in providing cellular energy. As recently reviewed by Montgomery and colleagues [7], the hypoxic pulmonary vasoconstriction (HPV) that redistributes the pulmonary perfusion and the activation of HIF-1α-mediated pathways that ameliorate the oxygen availability can override the regional hypoxia in healthy subjects. In the case of HAPE, hypoxia is not regional, and this leads to exaggerated HPV which may end up in overperfusion of the non-constricted pulmonary vessels [8] and eventually in pulmonary hypertension and edema. Currently, misdiagnosis is quite frequent due to the broad heterogeneity of clinical symptoms and the characterization of metabolites involved in the pathophysiology of HAPE would be of great value. 

### 2.2. Acute Respiratory Distress Syndrome

ARDS is an acute condition characterized by sudden onset of severe hypoxemia without evidence of heart failure or volume overload [9]. As it is a severe condition with multiple and complex etiology, it requires hospitalization in the Intensive Care Unit (ICU), and has a high mortality (35–46%) [10]. The main feature of ARDS is an increase in pulmonary capillary permeability. Protein-rich fluids accumulate inside the alveoli, as a result of the damage to the capillary endothelium and alveolar epithelium [11]. In several patients, ARDS is associated with low Hb despite persisting hypoxia, down to severe anemia (<8 g/dl Hb), which correlates with worse prognosis [12,13]. Frequent among ICU patients [14], this anemia may be due to inflammation.

### 2.3. Chronic Obstructive Pulmonary Disease

COPD is a prevalent airway disease functionally characterized by non or partly reversible proximal bronchial obstruction that is a major cause of respiratory disability [15]. Exposure to inhaled pollutants and most often to cigarette smoke [16] leads to chronic airway and lung inflammation that is regarded to promote structural changes, obstructions, and respiratory symptoms [17]. Small airways (inner diameter less than 2 mm) give the major contribution to airflow limitation in COPD since intraluminal obstruction could occur for mucus or substances deposition or for increased wall thickness [18]. Moreover, loss of alveolar attachment could alter the luminal diameter by means of small airway distortion [19]. Pulmonary vascular dysfunction, vascular remodeling, and pulmonary hypertension precede development of alveolar destruction, as seen in the lungs of a mouse model of emphysema and in lung tissue from humans with end-stage COPD [20].

Beyond small airway obstruction, pulmonary vascular abnormalities triggered by the inflammatory process may participate in COPD disability. Hypoxia-induced vascular changes may characterize the early onset as well as the disease evolution of COPD, contributing to pulmonary hypertension and heart dysfunction [21]. Due to hypoxia-mediated lung vessel remodeling, COPD patients exhibit structural alterations of the intimal layer in small pulmonary arteries [22], vessel wall thickening, proliferation of vascular smooth muscle, and infiltration of inflammatory cells [23]. 

Low levels of oxygen activate HIF-1α that controls the transcription of genes regulating angiogenesis, vascular remodeling, and glucose metabolism [24]. In response to HIF-1α, platelet derived growth factor β (PDGF β) is released in the endothelium, thus favoring the vasodilation of pulmonary arterial smooth muscle [25] together with vascular endothelial growth factor (VEGF), involved in tissue remodeling and angiogenesis in COPD [26].

### 2.4. Cystic Fibrosis

CF is a fatal genetic disorder caused by dysfunction of the anion transporter cystic fibrosis transmembrane conductance regulator (CFTR), which is expressed on the apical membrane of epithelial secretory cells. It involves several organs, but mortality is mainly due to lung disease. The reduced epithelial chloride transport and the excessive sodium reabsorption lead to dehydrated surface airway liquid, increased viscosity of the mucus layer, and plugging of the airway lumen [27,28]. Lumen obstruction caused by mucus plaques together with increased epithelial oxygen consumption probably due to enhanced ENaC-mediated sodium transport [27,29], create regional hypoxic niches within airway epithelial cells [30]. CF patients are characterized by perfusion deficit and the dysfunctional CFTR impairs the HIF-1α stabilization and activity thus affecting the adaptive response to hypoxia. The unresolved regional hypoxia exacerbates neutrophilic sterile inflammation that is triggered by the release of IL-1α. In fact, the hypoxic stimulus promotes the necrosis of airway cells and the consequent passive release of IL-1α together with the activation of NLRP3 inflammasome that in turn results in the active release of IL-1α [7].

## 3. The Variegate Pathways Involved in Hypoxia Adaptation

Hypoxia activates rho kinase, inducing vasoconstriction [31]. Hypoxic pulmonary vasoconstriction (HPV) is known as an adaptive mechanism that optimizes pulmonary ventilation-perfusion matching in regional hypoxia. Such phenomenon can be both protective and harmful, depending on the hypoxia condition to which the subject is exposed. In case of global hypoxia, as it happens at high altitude, or if the respiratory impairment involves most of the lung area, HPV can be harmful, as a potential cause of pulmonary hypertension [8,32]. On the other hand, when hypoxia is restricted to a confined area of the lung, HPV allows the blood flow to be deviated to the better ventilated compartments, in order to compensate the local lack of O_2_. The mechanism triggering HPV involves an impairment in the redox equilibrium that triggers the smooth cells’ contraction [8].

The main mechanism of adaptation involves the inducible transcription factors HIF-1α and HIF-2α, which enhance the transcription of several proteins with the aim of increasing oxygen availability [33]. One of these proteins, upregulated through HIF-2 is erythropoietin [34], which causes an increase in hematopoiesis and circulating Hb, as an attempt to optimize the transport of the available oxygen [35], Hb levels increase with altitude in acclimatized newcomers, but vary among high-altitude populations [36]. Higher Hb values are common among the Oromo population of East Africa, which lives at 4000 m above sea level, as compared to the values of a population living at lower altitude [37]. An excessive increase in hemoglobin can promote cardiovascular disease and pulmonary hypertension in those populations [38]. 

Recently, iron and SPL metabolisms have been considered as potentially involved in hypoxia adaptation response.

The relationship between hypoxia and iron metabolism has been widely explored as therapeutic targets for anemic patients [39]. Indeed, the PubMed hits for the terms “hypoxia” AND “iron” total more than 2500. A less explored field is the role of SPL in hypoxia (224 PubMed hits). This heterogeneous class of lipids plays, on the other hand, a key role in the inflammation enhancing hypoxia in respiratory diseases, and in hypoxia itself [40,41].

### 3.1. Hypoxia and Iron Metabolism: Role of Hepcidin

Since iron is an essential component of Hb, the response to hypoxia also increases the need for iron. In fact, HPV and the potential consequent hypoxic pulmonary hypertension may be reduced by iron supplementation and exacerbated in case of iron deficiency [42,43]. In order to optimize iron use for hematopoiesis, the degradation of ferroportin (Fpn) must be reduced. Fpn is the transmembrane protein that allows the release of iron from the cells, mediating its intestinal absorption, and subsequent release into the circulation, bound to its transporter, the transferrin. Fpn degradation is mediated by the regulating peptide hepcidin, whose decrease, in healthy subjects, contributes to a good compensatory response to hypoxia and to iron deficiency [44]. A decrease in hepcidin has indeed been observed in healthy subjects exposed to high altitude hypoxia, both after acute (hours) and chronic (weeks) exposure [45,46]. However, in the clinical setting, hepcidin is not yet considered among the routine parameters for the assessment of iron metabolism, although some studies are considering its decrease as a factor suggestive of the need for iron-based intravenous therapy [47]. The values described in literature are very variable and denote a marked difference between healthy subjects [48] or anemic subjects hospitalized in Intensive Care Unit, in which high values are observed [49].

As circulating hepcidin is mainly produced by the liver, most of the in vitro studies about hepcidin expression are performed on hepatic cell lines, such as HepG2. Hepcidin expression is enhanced by a treatment with the iron chelant deferoxamine [50], despite the main effect of such compound being a reduced iron availability. Interestingly, deferoxamine is also used in several in vitro studies as a hypoxia mimetic as it activates the HIF-1 α pathway [51]. This suggests that, other than an increased iron request, there are several mechanisms that play active roles in regulating hepcidin production and action on iron storage and use. HIF-2α expression in intestinal cells is influenced by a decrease in hepcidin expression other than by hypoxia. Inducible deletion of hepatic hepcidin in a mouse model has indeed been found to activate intestinal HIF-2α and rapid iron accumulation [52]. HIF2 inhibitors have been proposed to treat iron accumulation diseases characterized by dysfunction of the hepcidin/FPN axis [53].

#### 3.1.1. Iron Metabolism and HAPE

Hepcidin can be proposed as one of the potential markers of HAPE, as higher hepcidin values have been found in subjects developing HAPE, when compared to a group of subjects reaching the same altitude with the same timings, but without any sign of misadaptation [54].

#### 3.1.2. Iron Metabolism and ARDS

Anemic ICU patients, among which patients with ARDS, have higher values of hepcidin than healthy subjects [49]. In addition, ferritin, the protein involved in intracellular iron storage and now known as an acute phase protein, is increased in the plasma of ARDS patients [55]. Inflammation, particularly through the pro-inflammatory cytokine IL-6, is a cause of increased hepcidin production [50], which may therefore interfere with the previously described hematopoietic compensation mechanism.

#### 3.1.3. Iron Metabolism and COPD

In contrast to the physiological adaptation to hypoxia that should increase iron absorption and utilization with consequent compensatory Hb elevation [44], this pattern is observed only in a limited fraction of COPD patients: 40–50% of COPD patients instead develop iron deficiency, with anemia representing a predictive risk factor of worse outcome in 5–30% of cases [56,57]. Several studies demonstrated that COPD patients with chronic hypoxia have a reduced production of erythropoietin at either the level of the kidney or bone marrow. This response may correlate with an increase in systemic inflammatory markers though the interplay between iron, hypoxia, inflammation, and erythropoietin is complex and needs to be elucidated [58,59,60]. 

#### 3.1.4. Iron Metabolism and CF

Hypoxia maladaptation in CF has been correlated to altered iron homeostasis that comprises systemic iron deficiency as well as increased iron accumulation and defective iron sequestration in the airway cells. Furthermore, there is preliminary evidence that iron deficiency in CF patients prevents secondary erythrocytosis [61]. In in vitro CF airway epithelial cells, the deficiency of heme-oxygenase-1 (HO-1), which plays a pivotal role in regulating cellular iron, correlates with increased iron concentration [62]. This in turn impairs HIF-1α stability by promoting its prolyl hydroxylase-mediated polyubiquitination and proteasomal degradation leading to an altered cellular response to hypoxia [62]. 

In a study performed on a small cohort of CF patients with mild to moderate pulmonary dysfunction and borderline hypoxia [63], hemoglobin and hematocrit values did not significantly differ from normal control subjects while serum iron and iron-binding capacity were lower, leading to a tendency to anemia. A prospective observational study by Fischer et al. showed that a hypoxia driven increase in red cells mass is absent in CF patients who often exhibit normal or even decreased hemoglobin levels. The occurrence of subclinical anemia may involve the absolute iron deficiency due to gastrointestinal malabsorption, loss via sputum, a lack of vitamin E, and inflammation [61].

### 3.2. Hypoxia and Sphingolipid Metabolism 

SPLs are a minor class of lipids of all mammal cells composed by a hydrophilic head group protruding into the extracellular environment and a hydrophobic moiety, the ceramide (Cer), located into the membrane bilayer [64]. As the main components of plasma and intercellular organelle membranes, they have structural roles, but they also act as signaling molecules regulating cellular processes such as cell growth and death, senescence, inflammatory response [65,66]. Cer, the central hub of the SPL metabolism, is synthesized in the de novo pathway, from palmitoyl-CoA and serine, in the sphingomyelin (SM) hydrolysis pathway, which generates Cer from SMs and in the salvage pathway, from the catabolism of complex glycosphingolipids [67,68]. Cer can be cleaved by a ceramidase (CDase) to produce sphingosine (Sph) that, in turn, can be phosphorylated by two enzymes, sphingosine kinases 1 (SK1) and 2 (SK2) for the synthesis of sphingosine 1 phosphate (S1P) that is a crucial bioactive SPL.

Similar to iron, Cer, and S1P must be finely tuned to preserve cellular homeostasis. Several lines of evidence outlined the opposite cellular effects of Cer and S1P in response and adaptation to hypoxia. 

#### 3.2.1. Role of Ceramide

Cer levels increase in hypoxia and other forms of cell stress. Cer accumulated via activation of both de novo synthesis or SM hydrolysis pathway mediates hypoxia-derived cytotoxicity in different cellular models [69,70,71,72]. In neonatal mice exposed to 10% of oxygen (close to oxygen level at 5,260 m), cardiac Cer content was dependent on the acute (1 day) or chronic (1; 4; 8 weeks) hypoxia condition, suggesting a role for Cer decrease [73] together with its upstream precursor dihydroceramide increase [74] in the right ventricle adaptive response to chronic hypoxia. Such studies not only support a key role for Cer de novo synthetic pathway, but also propose the enzyme dihydroceramide desaturase which converts dihydroceramide to Cer as an important regulator in the adaptation to hypoxia [73,74]. Interestingly, this enzyme is oxygen-dependent [75]. Other evidence indicates that the augmented sphingomyelinase activity critically contributes to the hypoxia-induced increases in Cer content [76]. Klevstig and colleagues found that cardiac Cer accumulation was reduced in sphingomyelinase deficient mice under ischemic conditions [76]. In addition, Cer has been described as a promoter of hypoxic pulmonary vasoconstriction, although its vascular effects deserve further investigations [77]. These results would suggest that enhanced Cer synthesis and accumulation contribute in mediating the downstream toxic signals in response to hypoxia. 

Although S1P has intracellular targets, it acts commonly as an autocrine or paracrine mediator by binding to five cell surface S1P receptors [78]. In lung disease, S1P receptors 1 (S1PR1) are of particular interest since they enhance vascular barrier functions and they counteract apoptosis [79]. In a mouse model of emphysema induced by VEGF receptor blockade, Diab and colleagues reported that activation of S1P-S1PR1 signaling axis reduced the deleterious effects of ceramide on airspace enlargement [4]. Intracellular S1P accumulation, derived from exogenous S1P-activated SK1, prevents lung cell apoptosis by promoting the expression of HIF-1α and VEGF. This effect contributes to prevent the airspace enlargement together with the S1P-S1PR1 axis that maintains the alveolar septal integrity and mediate S1P effects on endothelial cell differentiation and integrity (together with S1PR3) via the Akt pathway [80]. In a model of fenretinide-induced emphysema, the protective effect of S1P injection was mediated by preservation of the transcription factor HIF-1α expression and its target gene VEGF and by the increase of Nrf2 expression [81].

#### 3.2.2. Role of Sphingosine 1 Phosphate

S1P is mainly considered a pro-survival and pro-proliferative factor. Since mature blood cells contain the biosynthetic machinery but not the degrading enzyme, they represent a large source of S1P for the plasma [82]. Intra erythrocyte S1P enhances the glycolytic metabolic fluxes leading to the generation of more 2,3-biphosphoglicerate that in turn promotes O_2_ release to protect against tissue hypoxia [82]. Human healthy volunteers brought to high altitude at 5260 m for up to 16 days, showed a time-dependent increase in plasma levels of S1P concurrently with elevated hemoglobin capacity to release oxygen [83]. Exogenous S1P has been proved effective as a potential preconditioning agent favoring adaptation to hypobaric hypoxia in in vivo models of different pathologies, such as respiratory, cardiovascular, and cerebral [84]. A pioneering study by Chawla et al. shows that S1P pre-treatment facilitates hypoxia adaptation in rats remained at 7620 m of altitude for 6 h. They propose that the beneficial effects of S1P rely on the enhanced blood oxygen carrying potential, mediated by HIF-1α stabilization and consequent dependent transcription of adaptive gene expression [85]. A recent work by Barbacini and colleagues investigated the potential role of SPL in metabolic adaptation of Andean children, born and living at 3775 m of altitude. They exhibit a prevalence of dyslipidemia and hypertension as compared to children at lower altitudes, suggesting that they undergo different metabolic adaptations. They observed that high density lipoprotein-cholesterol (HDL-C), Cer and S1P are involved in hypoxia adaptation though they propose S1P as the main actor in this process [86]. Further studies are needed to deepen in the complex mechanisms of SPL involvement in hypoxia adaptation though, up to now, several pieces of evidence would indicate S1P, instead of Cer, as a good candidate to counteract hypoxia-induced tissue damage.

#### 3.2.3. SPL Metabolism and HAPE

To the best of our knowledge, only one study reports the correlation between Cer and S1P alterations and HAPE. The metabolomics analysis of plasma metabolites from HAPE subjects and healthy controls by Guo and colleagues revealed that, among others, C-8 Cer and sphingosine were significantly higher in HAPE subjects [87]. The accumulation of these SPL metabolites could play a role in hypoxia maladaptation occurring in HAPE since it has been proven that Cer may contribute to pulmonary endothelial decreased barrier function, lung inflammation and edema [88]. Therefore, reestablishing the SPL homeostasis could represent a novel therapeutic target to improve acclimatization.

#### 3.2.4. SPL Metabolism and ARDS

Sphingolipids contribute to the modulation of endothelial barrier integrity [89,90]. In ARDS, endothelial barrier is disrupted and functionally altered. In a neonatal piglet ARDS model [91], aSMase hyper-activity and the consequent ceramide-C16/C18 accumulation in lung tissues correlate with inflammasome NLRP3 oligomerization, NF-κB and pro-fibrotic pathway activation. By inhibiting aSMase function with inositol 1,2,6-trisphosphate (IP3), Cer generation and NLRP3 oligomerization was completely blocked, resulting in improved oxygenation. Pandolfi and colleagues described a new aSMase-IL-6 pathway as pathogenic mediator of pulmonary vascular dysfunction in rat or human pulmonary artery smooth muscle cells (PASMCs) model of ARDS [92]. In a mouse model of ARDS, S1P analogue FTY720 reduces inflammatory lung injury induced by lipopolysaccharide (LPS) treatment [93]. Camp and coworkers synthesized novel FTY720 analogs and demonstrated that their effect in reversing the pulmonary vascular leak that characterizes ARDS in human pulmonary artery endothelial cells (HPAEC) is mediated by S1PR1-dependent receptor ligation [94]. Combination therapy of human umbilical cord (hUC-MSCs) and FTY720 in ALI/ARDS mice model induced by LPS resulted in higher survival rates and attenuated lung injuries [95]. 

#### 3.2.5. SPL Metabolism and COPD

Omics data on sphingolipid, arachidonic acid, hypoxia and energy signaling network in sputum of COPD patients, showed that upregulation of Cer, together with a dysfunctional response to hypoxia, strongly influence cellular energy metabolism. This is probably achieved by inhibiting mediators in energy metabolism and lipid trafficking such as fatty acid binding protein 4 and uncoupling protein 2 [96]. In COPD patients, oxygen limitation and airway inflammation correlate with sphingolipid imbalance whose main feature is the alveolar Cer accumulation [41,97]. Several pieces of evidence demonstrate that increased Cer further aggravates the compromised airway homeostasis [88]. Very recently, Bodas et al. showed that Cer/Sph ratio was elevated in COPD subjects as well as in mice exposed to cigarette smoke, worsening COPD-emphysema severity [98]. Excessive Cer content triggers damages of structural airway epithelial cells [99,100,101], which is involved in pulmonary tissue destruction [102] and contributes to maintain pulmonary inflammation [103].

A critical role for S1PR mediated signaling pathway has been demonstrated in the control of airway function in COPD though the precise mechanisms underlying the effects of S1PR modulation in COPD have to be further clarified. Cigarette smoke induced pulmonary S1P pathway upregulation in a mouse model of mild COPD, with an evident increase in S1PR2 and S1PR3 expressions [104]. Defective ability to phagocytose apoptotic cells in alveolar COPD macrophages has been related to the increased expression of the S1PR5 gene that correlates with its reduced methylation status [105]. On the contrary, the analysis of lung tissue samples from 25 patients with COPD showed that the relative mRNA expression of S1PR5 was reduced in COPD. Moreover, a positive correlation was found between the mRNA expression of S1PR5, S1PR1, and S1PR3, and between S1PR3 and S1PR2. In conclusion, the authors suggest S1PR5 as a possible novel target for pharmacotherapy [106]. S1PR1 has been found to be downregulated in human pulmonary diseases such as COPD [79] and induction of the S1PR1 receptor activity increases survival and vascular permeability [107].

#### 3.2.6. SPL Metabolism and CF

Though several in vitro and in vivo CF studies describe the involvement of Cer and S1P metabolism dysregulation in worsening the pulmonary conditions (for a comprehensive review, please refer to Aureli et al. [108]), the role of SPLs in mediating the adaptation to hypoxia is still a poorly investigated field. There is only one paper, by Tabelling et al. [40] that focused on the interplay between SPLs, hypoxia adaptation, and CF. The authors proposed a dual role for SPL signaling in CFTR-mediated HPV, suggesting an nSMase-derived Cer and SphK1-dependent conversion to S1P as the mediators of this hypoxia adaptation response. They demonstrated that, in CF, CFTR dysfunction impairs HPV by inhibiting the Cer-mediated translocation and the S1P-mediated activation of TRPC6 (transient receptor potential canonical 6) that plays a crucial role in the contraction of pulmonary artery smooth muscle cells [40].

## 4. Perspectives: Sphingolipid and Iron Metabolism in the Prognosis and Treatment of Chronic Respiratory Diseases

### 4.1. Sphingolipids and Iron Interplay 

Recently, SPL alterations in response to dysregulated iron pathways have been observed in different animal models and patients. As reviewed by Rockfield and colleagues [109], iron toxicity correlates with increased synthesis of SPLs in different eukaryotic organisms as well as in mammalian cell lines. 

**Figure 1:**
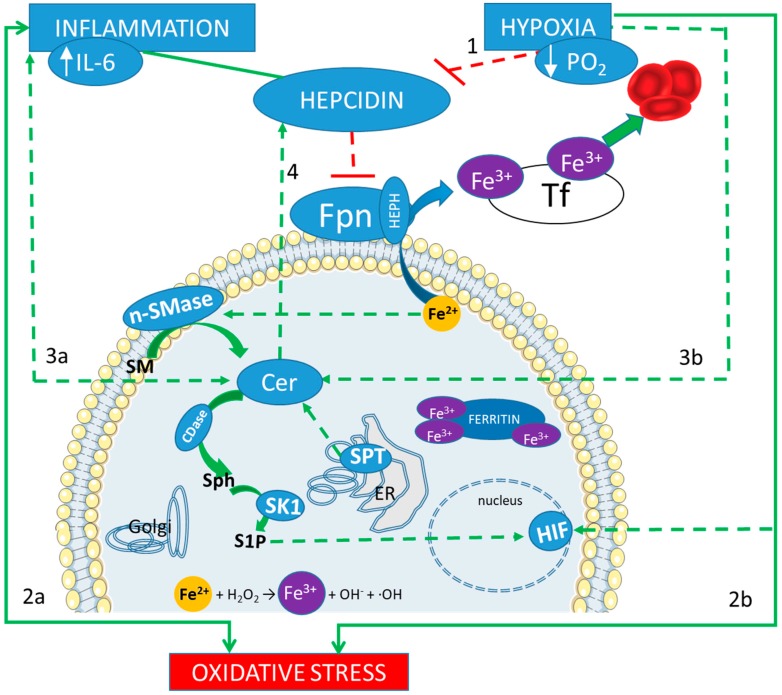
Iron and sphingolipids interplay in response to inflammation and hypoxia. A correct adaptation to hypoxia results in the inhibition of the regulator peptide hepcidin (line 1). Hepcidin main action is the reduction of the outflow of the intracellular ferrous iron (Fe^2+^), which is mediated by ferroportin (Fpn). Therefore, if Fpn is less inhibited, iron can be released in the blood stream, bound to the trasporter fransferrin (Tf) in its ferric form (Fe^3+^), and then reach the bone marrow, to contribute to the hematopoietic response. On the other hand, inflammation induces an increase in hepcidin, which blocks such adaptation. Both inflammation and hypoxia are sources of oxidative stress (lines 2a and 2b). An excess of intracellular iron can be a further source of oxidative stress, through the Fenton reaction (showed at the bottom). Both inflammation and hypoxia increase the production of Ceramide (Cer, lines 3a and 3b) derived by a de novo biosynthetic pathway, mediated by serin palmitoyl transferase (SPT) in the endoplasmic reticulum (ER), and by the hydrolysis of sphingomyelin (SM), mediated by neutral sphingomyelinase (nSMase). Cer accumulation promotes hepcidin expression (line 4) with a consequent increase in intracellular iron content, which, in turn, triggers Cer production (via activation of SM hydrolysis) in a vicious loop. Furthermore, ceramidase (CDase) converts Cer in sphingosine (Sph), which is phosphorylated by sphingosine kinase 1 (SK1) to produce sphingosine 1 phosphate (S1P). S1P acts as an oxygen-independent regulator of HIFs.

In *D. melanogaster* and *M. musculus*, iron-induced toxicity that recapitulates neurodegenerative damages seems to be mediated by SPLs and downstream signaling pathways [110]. In Neurodegeneration with Brain Iron Accumulation (NBIA) disease, a gene network analysis found an SPL enrichment as well as high iron levels in basal ganglia [111]. Iron accumulation triggers Cer production (via activation of SM hydrolysis) that in turn promotes hepcidin expression by transcriptional upregulation of HAMP gene (Hepcidin Antimicrobial Peptide) in HepG2 cells [112]. Thus, hepcidin entangles iron and Cer in a vicious loop that sustains the high levels of both mediators. Iron deficiency was found to reduce SPLs synthesis in *S. cerevisiae* strains, probably due to the inactivation of SPL biosynthetic enzymes that require iron as an essential cofactor [113]. Such interplay between iron and SPL, under hypoxia and inflammation conditions, is shown in Figure 1.

The inflammatory cascade, particularly through the pro-inflammatory cytokine IL-6, can increase hepcidin production [50], which may therefore interfere with the previously described hematopoietic compensation mechanism. Failure to regulate the mechanism of hepcidin decrease in response to hypoxia may limit the effectiveness of iron-based therapies or transfusions [49]. In fact, even a red blood cell transfusion has an inducing effect on hepcidin blood concentrations, in addition to increasing the concentration of free iron (non-transferrin-bound iron, NTBI), without however having effects on transferrin (Tf) saturation [114]. Tf, by binding iron, allows a reduction of toxicity and a more effective use by cells. Furthermore, its receptor (TfR) that allows the transport from the extracellular to the intracellular compartment increases in physiological response to iron deficiency. Tf saturation is often used for a more precise evaluation of the presence of iron in the blood, together with the total serum iron, which measures both the iron bound to transferrin, and therefore recruited for the hematopoiesis, and the NTBI. The increase in NTBI is one of the harmful effects of irregular iron metabolism as it can cause oxidative stress, catalyzing the formation of reactive oxygen species [2].

The link between iron/hepcidin content and SPL metabolism in inflammation is further strengthened since inflammatory hypoxia has been proved to modulate the synthesis of Cer and S1P and, in turn, to be modulated by these lipid molecules. Cer and S1P are both described as important signaling mediators in inflammation [115]. Cer accumulation induces inflammation [116] and hepcidin expression [112], while S1P acts as an oxygen-independent regulator of HIFs [117,118]. 

These data suggest that there is a unique relationship between SPL levels and iron-mediated cellular toxicity, since downregulating SPL metabolism is sufficient to allow survival in high iron conditions. Whether alterations in other elements of iron signaling pathway are induced in response to Cer and other SPLs is actually an open field. 

### 4.2. The Potential Prognostic Factors

Table 1 summarizes the main iron and sphingolipids metabolism markers and their role in influencing the adaptation to hypoxia. Further investigation on their role in determining the respiratory diseases prognosis are still required, especially for ceramide and intra-erythrocyte S1P. Here, we propose to compare the adaptation to high altitude in healthy subjects to the one to respiratory disease, in order to propose new biomarkers. Ceramide, measurable in plasma samples through mass spectrometry, has already been proposed as a biomarker of pulmonary and hepatic metastasis response to specific radiochemotherapy [119]. A metabolomic study of plasma shows that ceramides, sphingomyelin, and gangliosides levels are altered in specific COPD phenotypes such as emphysema and in COPD exacerbations [120]. Cystic Fibrosis is the first disease in which excessive airway epithelial cell ceramide was found to play a role in airway pathophysiology [116,121,122]. In the lung parenchyma, the accumulation of ceramide but also the ceramide to S1P balance influences cell fate and lung remodeling responses. High ceramide-to-S1P ratio due to either ceramide increase [4] or S1P decrease [123] correlates with emphysema-like pathology and cell death.

### 4.3. Enhancing Hypoxia Adaptive Mechanism for the Treatment of Inflammatory Anemia

HIF and hepcidin are both already considered as therapeutic targets for inflammatory anemia, also in subjects without any respiratory disease [39]. The clinical utility of enhancing the HIF pathway to increase and optimize hemopoiesis is already under assessment through phase II clinical studies. Roxadustat, a small molecule which inhibit HIF prolyl-4-hydroxylases, therefore increasing HIF action, has been showing its effectiveness in patients with chronic kidney disease, and, as a secondary effect, was found to decrease circulating cholesterol [131]. Lexaptepid, an L-oligoribonucleotide with a strong affinity to hepcidin mRNA, has been assessed in healthy subjects for its ability of decreasing hepcidin levels [132], and might be useful in treating anemic COPD patients too. On the other hand, a decrease in hepcidin has been suggested as a potential criteria to discriminate the patients who may need iron-based intravenous therapy [47]. Iron infusion has an acute increasing effect on hepcidin concentration, but has been shown to reduce the hypoxia-induced increase in Pulmonary Artery Systolic Pressure [43]. 

Although the ubiquitous SPL expression could be a limitation, there are ongoing preclinical and clinical studies targeting both Cer and S1P in CF [133,134]. S1PR1 agonists may represent a new therapeutic strategy in the treatment COPD [4]. As reviewed by Becker and colleagues [133,134], systemic treatment with amitriptyline, an inhibitor of acid sphingomyelinase, is used in clinical trials to improve lung function in CF patients. Moreover, the application of recombinant acid ceramidase, a Cer degrading enzyme, is currently under development for clinical use in children with Farber disease, and it could be evaluated in CF patients since it has been proven to prevent infection in CF mice.

## 5. Conclusions

Among the various mechanisms involved in the patho-physiological adaptation to hypoxia, iron and SPL pathways play a key role, as described in pathological conditions such as HAPE, ARDS, COPD, and CF. Hepcidin, Cer, S1P, and their interplay in hypoxia are raising growing interest both as prognostic factors and therapeutical targets.

## Figures and Tables

**Table 1:** Iron and SPL metabolism markers and their trend in comparison to the normal values, in response to different hypoxic conditions.

Parameter	High Altitude Good Adaptation	High Altitude Bad Adaptation (HAPE)	ARDS	COPD	Cystic Fibrosis
hepcidin	Low [44,45,46]	High [54]	High (anemic ICU patients [49])	Low in stable [57,124] high in exacerbations [125]	Low in stable kids [126]
ferritin	Low [44,45]	Normal/high [54]	High [55]	Normal/high [56]	Normal/high [61]
Erythropoietin (EPO)	High [44,45]	High [127]	?	high [56] low in exacerbations [128]	Normal/high [61]
hemoglobin	High [35]	Very high/low [44,45]	Low (ICU patients) [14]	high [128] Low in worse prognosis [128]	Normal/Low [61]
SPL Metabolites	S1P high [86]	Cer High [87]	S1PR3 high [129] and SMase high [92] in worse prognosis	Cer High [98,130]	Cer high [121]

## Abbreviations

CDase	ceramidase
Cer	ceramide
CF	Cystic Fibrosis
CFTR	Cystic Fibrosis Transmembrane conductance Regulator
COPD	Chronic Obstructive Pulmonary Disease
Fpn	ferroportin
HAPE	High altitude pulmonary edema
Hb	hemoglobin
HIF	Hypoxia-inducible factor
HPV	Hypoxic Pulmonary Vasocostriction
nSMase	neutral sphingomyelinase
O2	oxygen
S1P	sphingosine 1 phosphate
Sph	sphingosine
SPL	sphingolipids
